# Liquid Helium Enhanced Vitrification Efficiency of Human Bone-Derived Mesenchymal Stem Cells and Human Embryonic Stem Cells

**DOI:** 10.3390/bioengineering8110162

**Published:** 2021-10-26

**Authors:** Mengjia Dou, Chennan Lu, Jing Liu, Wei Rao

**Affiliations:** 1 CAS Key Laboratory of Cryogenics, Technical Institute of Physics and Chemistry, Chinese Academy of Sciences, Beijing 100190, China; doumengjia17@mails.ucas.edu.cn (M.D.); luchennan18@mails.ucas.edu.cn (C.L.); jliu@mail.ipc.ac.cn (J.L.); 2 School of Engineering Science, University of Chinese Academy of Sciences, Beijing 100049, China; 3 Beijing Key Laboratory of Cryo-Biomedical Engineering, Beijing 100190, China; 4 School of Future Technology, University of Chinese Academy of Sciences, Beijing 100049, China

**Keywords:** liquid helium, liquid nitrogen, vitrification, stem cells

## Abstract

Stem cells have the capacity to self-renew and differentiate to specialized cells, which are usually sensitive to cryopreservation. Therefore, the cell survival rate of stem cells using common cryopreservation protocol is generally not ideal. High cooling rates are crucial for decreasing the usage of cryoprotectants (CPAs) and promoting the successful vitrification of stem cells. In this study, we adopted liquid helium (LHe) instead of liquid nitrogen (LN_2_) as the cryogen to achieve high cooling rates for vitrifying stem cells with high viability and complete functions. A numerical model was established to simulate the cooling processes of vitrifying specimens by immersing them in LHe and LN_2_. The calculated results revealed higher cooling rates when plunging specimens into LHe than into LN_2_. The high viability of human bone-derived mesenchymal stem cells (hBMSCs) and human embryonic stem cells (hESCs) after vitrifying into LHe also shows the superiority of LHe as the cryogen. Furthermore, considerable cell viability was achieved by vitrification in LHe, even when decreasing the concentrations of CPAs. Additionally, post-vitrification, the cells still maintained high attachment and proliferation efficiency, normal stemness, and multipotential differentiation both for hBMSCs and hESCs. LHe is prospective to be employed as a universal cryogen for vitrification which has a great potential for widespread applications, including bioengineering and clinical medicine.

## 1. Introduction

Stem cell-based therapy which restores tissue structures and functions is a floushiring area in modern medicine [1]. Especially, mesenchymal stem cells with immunomodulatory and immunosuppressive properties are ideal for allogeneic transplantation [2]. Human embryonic stem cells (hESCs) show excellent advantages in tissue regeneration and transplantation [3]. To follow the therapy schedule, large quantities of stem cells need to be preserved with high cellular viability and excellent functions to achieve with time. The continuous culture is expensive and time-consuming, and stem cells may lose the stemness and functions during the processes. Cryopreservation ensures the suspension of chemical, biological, and physical processes of cells at ultra-low temperatures for the long term [4], which is an important supporting technology for stem cell-based therapy [5].

There are usually two kinds of methods for cell cryopreservation: slow freezing and vitrification [6]. Cells cryopreserved by slow freezing must suffer from ice damage and the process is time-consuming and complex. Recently, vitrification, which converts liquid water into a glass-like amorphous solid without any ice damage, shows great potential in cell cryopreservation [7]. However, current vitrification can only be achieved by rapid cooling rates and high concentrations of cryoprotectants (CPAs) to suppress the possibility of ice formation. High CPAs cause osmotic damage and toxicity to cells, including hemolysis, neurotoxicity, respiratory arrest, cardiovascular failure, and fatal arrhythmias [8,9]. Therefore, numerous researchers contribute to discovering biocompatible and high-efficiency CPAs [10,11,12] or new cooling and warming methods to decrease the usage of traditional CPAs [13,14,15,16].

Apart from decreasing volumes of cryopreserved specimens and using carrier materials with high thermal conductivity [17,18], a cryogen with lower temperature and higher heat transfer efficiency will increase cooling rates greatly to promote post-cryopreservation survival [19,20]. Liquid nitrogen (LN_2_) (−196 °C) is a widely used cryogen for cell vitrification. Recently, with the development of the cryogenic industry, it is easier to obtain liquid helium (LHe) (−269 °C) [21]. The larger temperature difference between LHe and a vitrified sample shows the potential for a higher cooling rate [16]. For these reasons, LHe was proposed to be the vitrification cryogen in recent years as a way to improve cooling rates and survival of vitrified cells [16,20,22].

Herein, we established a numerical model to simulate cooling processes of vitrified samples immered in LN_2_ or LHe and calculated cooling rates theoretically. Then, we vitrified human bone-derived mesenchymal stem cells (hBMSCs) and hESCs using LN_2_ or LHe, respectively, to determine the vitrification efficiency of these two cryogens experimentally. The thermodynamic process of cell vitrification using LN_2_ and LHe as cryogens were analyzed to present the advantage of LHe as a cryogen. Finally, the cell viability and functions were checked to testify the validity and security of LHe as the cryogen.

## 2. Materials and Methods

### 2.1. Physical Model 

**Figure 1:**
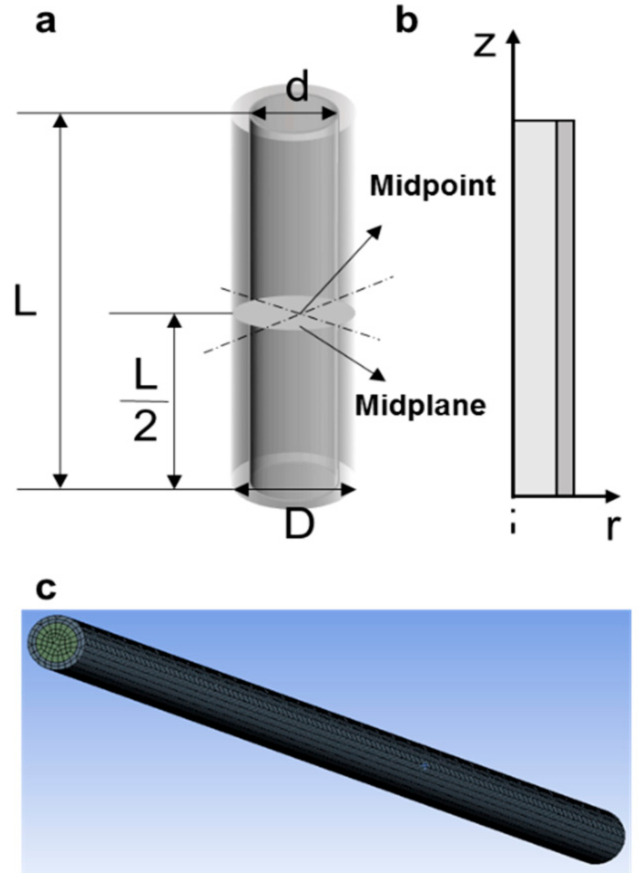
The straw model is used in vitrification. (**a**) A 3-D model of vitrification straw. (**b**) Bidimensional revolution surface. (**c**) Finite element meshes of the straw model.

A commercial polypropylene straw (L = 130 mm, D = 2.6 mm, d = 1.9 mm) (Figure 1) was used to vitrify cells. The model system contained two concentric cylinders: the inner cylinder was filled with cell suspensions and the outer cylinder was polypropylene [23]. The physical properties were previously described [24]. When the straw was immersed in LN_2_ or LHe, film boiling occurred. Heat is transferred by conduction through the straw wall, and then into the cell suspension inside.

The heat transfer problems were solved by the following equations:

(1) $$\rho Cp\frac{\partial T}{\partial t}r=\frac{\partial }{\partial r}\left(kr\frac{\partial T}{\partial r}\right)+\frac{\partial }{\partial z}\left(kr\frac{\partial T}{\partial z}\right)$$

where ρ is the density, Cp is the specific heat, t is the cooling time (t>0), k is the thermal conductivity, while r and z are the radials and axial coordinates.

Initial condition:

(2) $$T=T_{0}\;\mathrm{at}\;t=0$$

where $T_{0}=4\;{}^{\circ}C$ is the initial temperature for all materials. 

Boundary conditions:

(3) $$-k\left(\nabla T\cdot n\right)=h\cdot \left(T-T_{ext}\right)$$

where n is the external normal unit vector, $T_{ext}$ is the external temperature of LN_2_ or LHe, and h is the surface heat transfer coefficient. h for plastic straws immersed in LN_2_ and LHe has not been reported. h for straws plunged into LN_2_ was assumed to be in the range of 125–1000 W/(m^2^∙K) according to the previous report [19], and h for straws plunged in LHe was assumed in the range of 167–2000 W/(m^2^∙K) here [25] using the same assumption in the literature [19].

Commercial software Ansys Fluent was applied to solve the heat transfer problems. Vitrification without the latent heat of ice crystallization during cooling was set. Therefore, all materials were assumed to maintain constant thermal properties [23]. The cooling rate (°C/min) was calculated by the time needed to reduce from 4 °C (the initial core temperature of cell suspensions) to −90 °C (avoiding ice formation) at the warmest point of the model system [23].

### 2.2. Cell Culture 

The hBMSCs (SCSP-405, purchased from Stem Cell Bank, Chinese Academy of Sciences) were fed with hBMSCs’ complete growth medium (Cellapy, Beijing, China) in a humidified incubator. The medium was changed thrice per week. When reaching 70–80% confluency, hBMSCs were detached for passage or experimental usage. 

The hESCs (SCSP-302, purchased from Stem Cell Bank, Chinese Academy of Sciences) were cultured in Pluripotency Growth Master 1 (Cellapy, Beijing, China) and culture medium was changed each day. hESCs were propagated when reaching 70–80% by a 1:4. 

### 2.3. Vitrification

The collected hBMSCs were firstly suspended in the equilibration solution (EFS20, EFS17.5, and EFS15, Table 1) for 5 min and then mixed with 50 μL vitrification solution (EFS40, EFS35, and EFS30) at a density of 5 × 10^6^ cells mL^−1^. For hESCs, the equilibration (EDS20, EDS17.5, and EDS15) and vitrification (EDS40, EDS35, and EDS30) solutions were changed as shown in Table 1. Subsequently, the cells were immediately plunged into LN_2_ or LHe with 0.25 mL straw (Minvitro, Guangzhou, China) for vitrification. For cells vitrified in LHe, they were transferred into LN_2_ for long-term storage. When rewarming cells, the straws containing hBMSCs or hESCs were rapidly immersed in a 0.2 M trehalose (BEIJINGSHIJI, Beijing, China) bath at 37 °C. Finally, the cells were successively suspended in 5 mL 0.5 M trehalose, 5 mL 0.25 M trehalose, and a complete culture medium for 5 min for further analysis.

### 2.4. Immediate Cell Viability

The immediate cell viability was evaluated by the Live/Dead viability kit (Solarbio, Beijing, China), containing cell-permeant Calcein AM marking live cells with green fluorescence and Ethidium homodimer-1 marking dead cells with red fluorescence. Once thawed, the cells were re-suspended with the Live/Dead viability kit for 30 min in a humidified incubator according to the manufacturer’s instruction. Then, at least 3 fluorescence images for each sample were taken using a camera (LSM710, Zeiss, Jena, Germany) to count the cell viability.

### 2.5. Attachment Efficiency

The thawed cells were seeded in 96-well plates with 4000 cells per well. After 3 days, cells were incubated for 3 h at 37 °C with 110 μL medium containing 100 μL complete growth medium and 10 μL CCK-8 solution (Solarbio, Beijing, China). Finally, the optic density values at 450 nm were measured by a Microplate Reader (MultiSkan FC, Thermo Scientific, Waltham, MA, USA) to quantify live cell numbers. The attachment efficiency was calculated according to CCK-8 protocols.

### 2.6. Morphology and Proliferation

Once thawed, the cultured cells were observed with a microscope (SIM, Zeiss, Jena, Germany) and captured at 1, 2, and 3 days to obtain their morphology. For evaluating proliferation, the fresh and thawed cells were cultured at 4000 cells per well in 96-well plates. After 1, 2, and 3 days, the cells were rinsed with DPBS twice and their numbers were evaluated by CCK-8 kit solution using the methods described above. The proliferation of fresh and thawed cells was calculated by quantifying the relative cell numbers of days 2 and 3 to that of day 1.

### 2.7. Flow Cytometry Analysis

Expressions of four surface antigens CD45^high^, CD90^high^, CD34^low^, and CD44^low^ of fresh and thawed hBMSCs were analyzed using flow cytometry (BD LSRFortessa, Franklin Lakes, NJ, USA). For hESCs, SSEA-3^high^ and TRA-1-81^high^ (Biolegend, San Diego, CA, USA) were tested. The harvested cells were incubated with antigens (FITC anti-human CD44, APC anti-human CD34, PE anti-human CD90, FITC anti-human CD45 for hBMSCs, PE anti-human/mouse SSEA-3 and PE anti-human TRA-1-81 for hESCs) (BioLegend, San Diego, CA, USA) separately at 4 °C for 30 min according to the manufacturer’s instructions. After getting rid of unbound antigens by rinsing cells in cell stain buffer, the cells were analyzed by flow cytometry (BD LSRFortessa, Franklin Lakes, NJ, USA) and FlowJo software to detect surface antigen expression.

### 2.8. Immunofluorescence Staining

Non-vitrified and vitrified hBMSCs were tested surface antigens CD31^low^ and CD44^high^ (Abcam, Cambridge, UK) (Table 2) by immunofluorescence staining. hESCs were stained with four antibodies against pluripotent marks: Oct-4^high^, SOX2^high^, SSEA-4^high^, TRA-1-60^high^ (Table 2). Immunofluorescent staining was performed as follows: Firstly, cells were fixed with 4% paraformaldehyde for 90 min at room temperature when growing to 30~60% confluency. For intranuclear antigens, Oct-4 and SOX2, cells should be permeabilized in 0.5% Triton X-100 for 15 min and blocked in 1% bull serum albumin for 30 min. Then, cells were incubated with primary antibodies: CD31 and CD44 for hBMSCs, Oct-4, SOX2, SSEA-4, and TRA-1-60 for hESCs (1:100 diluted in 3% BSA) overnight at 4 °C in the dark. Subsequently, cells were incubated in corresponding secondary antibodies (1:100 dilution) at room temperature for 60 min in the dark. Later, cell nuclei were stained in 4′,6-diamidino-2-phenylindole (DAPI, Beyotime, Haimen, China) for 10 min in the dark. Finally, cells were observed with SIM (Zeiss, Oberkochen, Germany).

### 2.9. Reverse Transcriptase Quantitative Polymerase Chain Reaction (RT-qPCR) Analysis

The total RNA from fresh and thawed hBMSCs and hESCs were purified using the RNeasy^®^ Mini Kit (Qiagen, Hilden, NRW, USA) according to the manufacturer’s instructions. After measuring the RNA concentration of each sample by the spectrophotometer (NanoDrop^TM^ One, Thermo Scientific, Waltham, MA, USA), the RNA of each sample was reverse-transcribed into cDNAs using the iScriptTM cDNA synthesis kit (Bio-Rad, Hercules, CA, USA) by T100^TM^ Thermal Cycler (Bio-Rad, Hercules, CA, USA). Later, primers (Table 3), template cDNAs, and Power SYBR^®^ Green PCR Master Mix (Thermo Scientific, Waltham, MA, USA) were applied to determine the expression of mRNA using the CFX96^TM^ Real-Time System (Bio-Rad, Hercules, CA, USA). Finally, gene expression was calculated with the ΔΔCt method [26]. For hBMSCs, three stem genes were assessed: PPARγ, Collagen I, Sox9. Glyceraldehyde 3-phosphate dehydrogenase (GAPDH) is the housekeeping gene. For hESCs, Oct-4, NANOG, SOX2, and GAPDH (housekeeping gene) were assessed.

### 2.10. Differentiation Capacity of hBMSCs

After vitrification, the multilineage differentiation potential of hBMSCs (adipogenic, osteogenic, and chondrogenic differentiation) was tested according to Cyagen (Santa Clara, CA, USA) protocols. For adipogenic differentiation, when cells grew over confluency, an adipogenic induction medium was supplied to culture cells for 3 days, and an adipogenic maintenance medium was added for 1 day. After 4 cycles of induction and maintenance, only an adipogenic maintenance medium was supplied for 5 days to ensure the growth of lipid droplets. After induction and maintenance, cells were fixed with 4% paraformaldehyde solution at room temperature for 30 min and then stained by Oil Red O working solution for 30 min. Finally, cells were observed as lipid droplets marked with Oil Red O working solution using a microscope. For osteogenic differentiation, cells were cultured in 0.1% collagen-coated plates until 60~70% confluency. Subsequently, an osteogenic induction medium was added to culture hBMSCs for 4 weeks. Similarly, hBMSCs were fixed and stained calcific deposition by Alizarin Red S working solution for 5 min. To induce chondrogenic differentiation, 0.5 mL hBMSCs suspension at a concentration of 5 × 10^5^ cells mL^−1^ was transferred to a 15 mL polypropylene tube and centrifuged to form pellets. A chondrogenic medium was used to induce cell differentiation for 28 days. Later, pellets were also fixed and tissue sections were embedded by paraffin. After deparaffinization, sulfated proteoglycan deposits of induced cells were stained by alcian blue solution for 30 min for observation. To analyze quantificationally differentiation results, at least 10 images of differentiation were conducted by IMAGE J. 

### 2.11. Karyotype Analysis of hESCs

For hESCs karyotype analysis, cells after vitrification in LHe were cultured and then incubated in a medium containing 50 ng/mL colcemid for 6–8 h at 37 °C. Later, cells were trypsinized, re-suspended in KCl, incubated at 37 °C for 20~40 min, and fixed in 3:1 methanol: acetic acid. hESCs were spread on glass slides and stained with Giemsa solutions for 5 min. Finally, a standard G-banding technique was applied and the chromosome pair software (VideoTesT-Karyo 3.1) was used to detect karyotype.

### 2.12. Teratoma Formation

Teratoma formation was performed in 4-week-old male severe combined immunodeficient (SCID) mice (CB17/Icr-*Prkdc*^scid^/IcrlcoCrlVr, Vital River, Beijing, China) to test pluripotency of vitrified hESCs. After 3 passages were propagated, approximately 1 × 10^7^ vitrified cells were injected subcutaneously into four SCID mice. Non-vitrified hESCs were induced to form teratoma in parallel as the control sample. Then, 2~3 months later, teratomas were fixed in 4% paraformaldehyde for 24 h, embedded in paraffin, and stained using hematoxylin and eosin to perform histological analysis. 

### 2.13. Statistical Analysis

All experiments were performed at least three times and the data were presented as mean ± standard deviation. The student’s t-test was used to determine statistical significance. A *p*-value < 0.05 was considered statistically significant.

## 3. Results and Discussion

### 3.1. Theoretical Prediction of Cooling Rates of Vitrification Straws Plunged into Liquid Nitrogen or Helium

**Figure 2:**
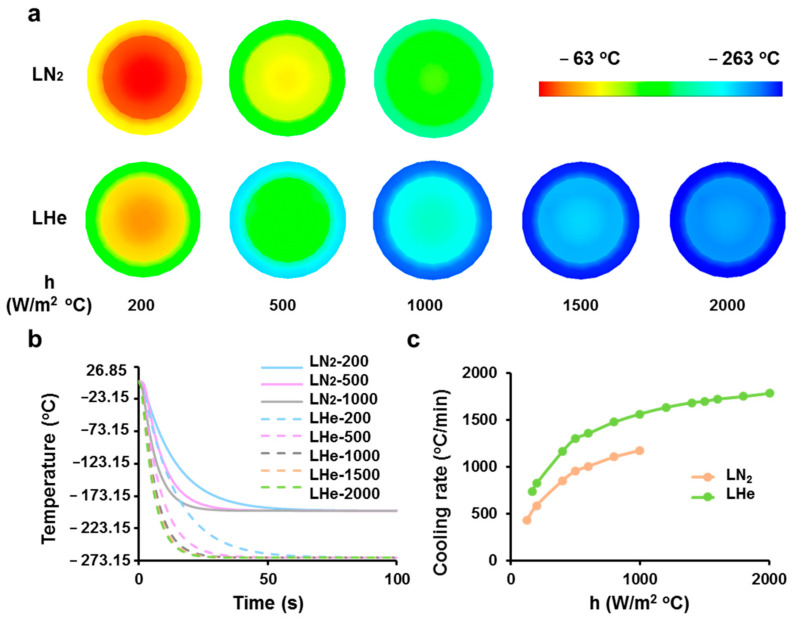
Theoretical prediction for cooling processes of vitrification straws plunged into LN_2_ or LHe. (**a**) Temperature contours of the midplanes of water-filled straws after immersing into LN_2_ or LHe for 3.2 s at different heat transfer coefficients. (**b**) Thermal histories of the midpoints of cell-suspension-filled straws immersed into LN_2_ or LHe at different heat transfer coefficients. (**c**) Effect of heat transfer coefficients on cooling rates at the midpoints of cell-suspension-filled straws from 4 °C to −90 °C when plunged into LN_2_ or LHe.

The transient temperature distributions of vitrification straws immersed into LN_2_ or LHe at various heat transfer coefficients were calculated by numerical models. For the straw model immersed in LHe with a larger heat transfer coefficient, it was cooled faster (Figure 2a,b). The temperature of its midpoint (the warmest point in the water-filled straw) reached −90 °C at 3.2 s after immersion. The temperature contours at 3.2 s were presented to compare temperature distributions with different heat transfer coefficients both for LN_2_ and LHe (Figure 2a). As shown in Figure 2a,b, even with the same heat transfer coefficients (200, 500, and 1000 W/m^2^∙°C), the temperatures of straws decreased more quickly in LHe than that in LN_2_. For both LN_2_ and LHe, the cooling rates increased quickly with the increase of heat transfer coefficients but then increased slowly (Figure 2c). This indicated that even with larger heat transfer coefficients, the cooling rates would not increase greatly for the limited heat conductivity of straws. In conclusion, the numerical model results illustrated that cell suspensions vitrified in LHe were cooled faster than that in LN_2_.

### 3.2. Vitrification in Liquid Helium Assisting Survival of Cells

**Figure 3:**
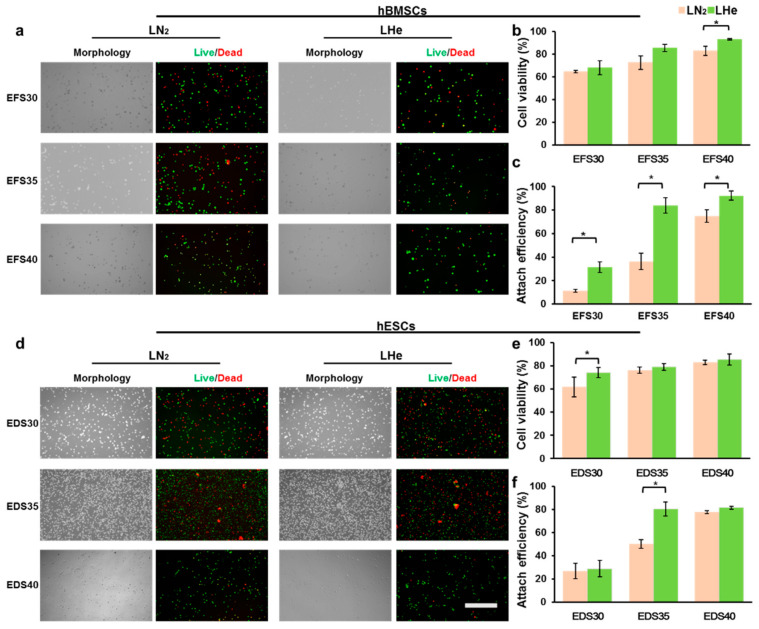
Survival rates of hBMSCs and hESCs after vitrification in LN_2_ and LHe with different concentrations of CPAs. (**a**) Morphology and fluorescence micrographs of live (green) and dead (red) hBMSCs after vitrification in EFS30, EFS35, and EFS40. (**b**) Immediate quantitative cell viability of hBMSCs after vitrification. (**c**) Attach efficiency of hBMSCs after vitrification. (**d**) Morphology and fluorescence micrographs of live (green) and dead (red) hESCs after vitrification in EDS30, EDS35, and EDS40. (**e**) Immediate quantitative cell viability of hESCs after vitrification. (**f**) Attach efficiency of hESCs after vitrification. Scale bar: 200 µm. *: *p* < 0.05.

Based on the results of the numerical simulation above, we vitrified cells to test the advantage of LHe as the cryogen in further (Figure 3). To guarantee that the cryopreservation results reflected a universal biophysical phenomenon rather than a specific response of one kind of cells or CPAs to LHe, two kinds of classical vitrification solutions (EFS40 and EDS40, see Table 1) were used to vitrify two kinds of sensitive cells, hBMSCs and hESCs, respectively. After vitrification, there was a significant difference in immediate cell viability between hBMSCs vitrified in LN_2_ (82.6 ± 4.1%) and LHe (93.0 ± 0.7%) (Figure 3a,b). However, LHe cryopreservation did not assist the cell viability of hESCs greatly (83.0 ± 1.9% vs. 85.4 ± 4.9%) (Figure 3d,e). Moreover, the attachment efficiency after three days was consistent with the cell viability both for hBMSCs and hESCs (Figure 3c,f). Then, the concentrations of CPAs were decreased from 40% to 30% to test the cryopreservation efficiency of LN_2_ and LHe (Table 1). For hBMSCs, though the cell viability was not impacted after being vitrified in LHe (Figure 3b), the attachment efficiency was increased significantly both for EFS30 and EFS35 groups (Figure 3c). For hESCs, the cell viability in the EDS30 group increased greatly. The attachment efficiency in the EDS35 group also increased significantly by replacing LN_2_ with LHe as the cryogen.

**Figure 4:**
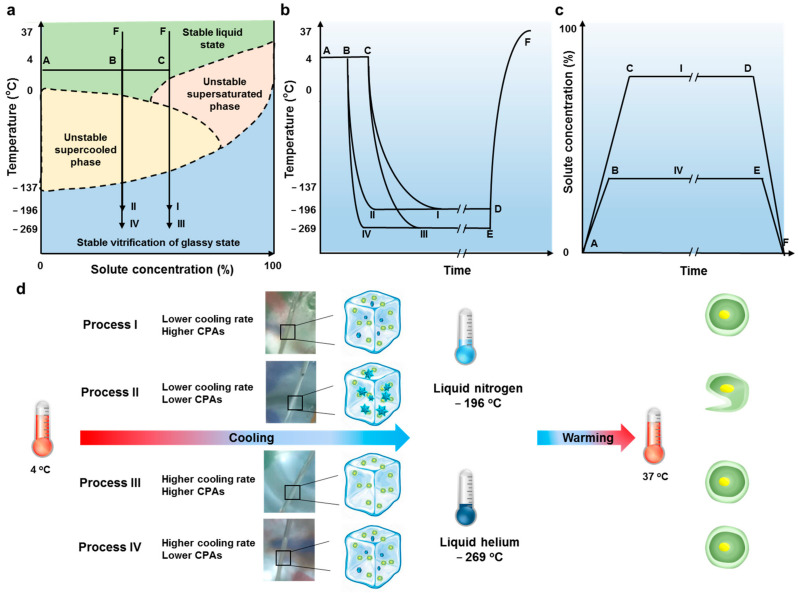
Thermodynamic process analysis of cell vitrification. (**a**) Phase diagram of biospecimens processed using different vitrification methods during cooling processes. Arrows indicate the direction of the preservation process. (**b**) Thermal time course. The latent heat of phase transitions was neglected both for cooling (heat release) and warming (heat absorption) processes. (**c**) Time course of the solute concentration. I was the optimal process for LN_2_ as the cryogen, IV was the optimal process for LHe. Long-term storage was presented by ‘//’ in (**b**,**c**). (**d**) Illustration for cell vitrification using liquid nitrogen or liquid helium as cryogens.

Stem cells could be preserved for long-term cryopreservation at stable glassy states [27]. However, the high concentrations of CPAs or high cooling rates were requested to enter into glassy states (Figure 4a–c). For high concentrations of CPAs, stem cells could be vitrified with high cell viability both using LN_2_ (Process I: A → C → I → D → F) and LHe (A → C → III → E → F) as cryogens. For low concentrations of CPAs, stem cells could only be vitrified successfully using LHe (Process I: A → B → IV → E → F). Process II (A → B → II → D → F) holding low cooling rates and low concentrations of CPAs could not vitrify stem cells. According to the evaluation of cell viability and attachment efficiency, the high cooling rate using LHe as the cryogen contributed to the survival of cells in vitrification (Figure 4d). Moreover, the high rate was beneficial to reduce the usage of CPAs, which hold biotoxicity (Figure 4c,d). Consequently, these in vitro experiments with hBMSCs and hESCs are in great agreement with results in the modeling studies above: the LHe as the cryogen provided faster cooling rates for vitrified samples.

### 3.3. Maintenance of Cell Morphology and Proliferation after Vitrification in Liquid Helium

**Figure 5:**
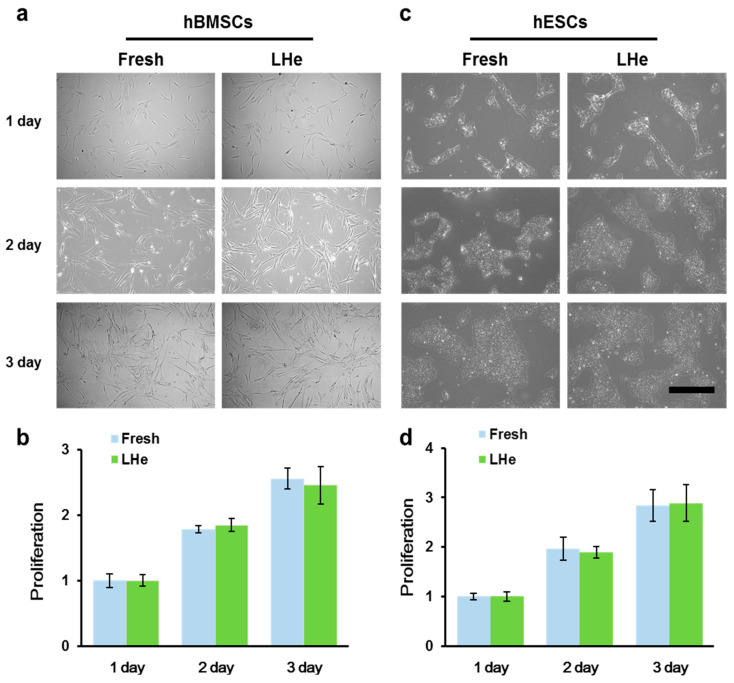
Cell morphology and proliferation were not impacted post vitrification. (**a**) Morphology and (**b**) proliferation of fresh and vitrified hBMSCs. (**c**) Morphology and (**d**) proliferation of fresh and vitrified hESCs. There was no significant difference in proliferention every day between group Fresh and LHe both for hBMSCs and hESCs. LHe: vitrification in LHe. Scale bar: 200 µm.

The high cell viability and attachment efficiency post vitrification even with low concentrations of CPAs showed the potential of LHe as the cryogen of cell vitrification, but it remains necessary to test the long-term viability and functional properties to ensure its biocompatibility. The long-term viability was assessed by both cell attachment efficiency and proliferation. For hBMSCs vitrified in LHe, the spindle fibroblastic morphology was similar to that of fresh hBMSCs during culturing for 3 days (Figure 5a). Furthermore, there was no significant difference in proliferation between fresh and vitrified hBMSCs (Figure 5b). hESCs also retained undifferentiated colony morphology after vitrification in LHe (Figure 5c). Additionally, cell proliferation was not affected (Figure 5d). These data indicated the vitrified hBMSCs and hESCs survived well three days after being vitrified in LHe.

### 3.4. Stemness and Multilineage Differentiation of hBMSCs Vitrified in Liquid Helium

**Figure 6:**
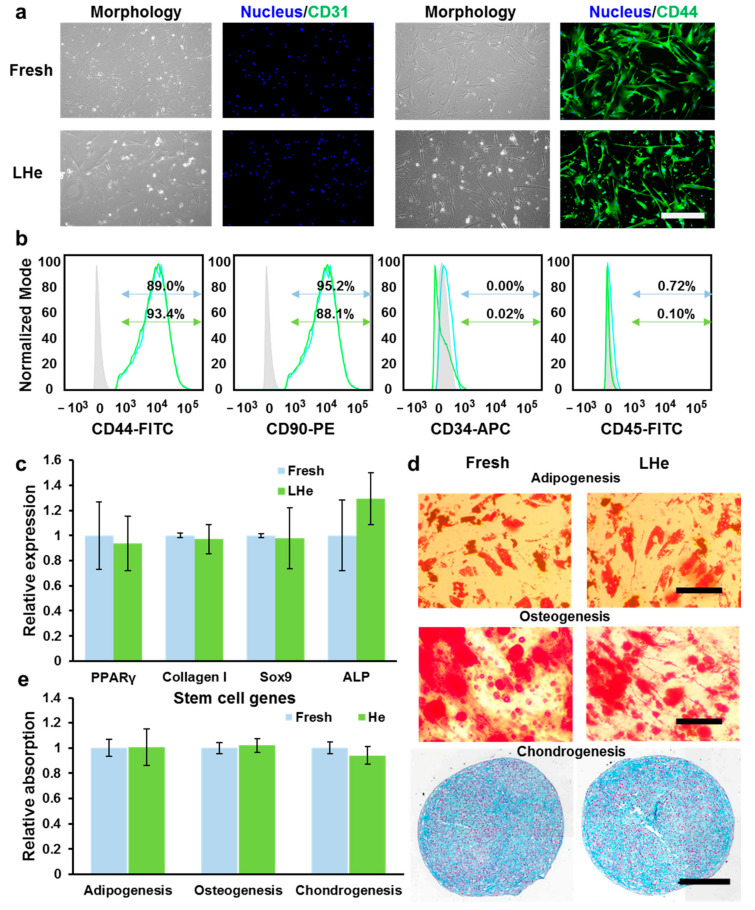
The hBMSCs retained stemness and differentiation ability after vitrification in LHe. (**a**) Immunofluorescence staining of vitrified hBMSCs showed the high expression of stem cell markers CD44 and the low expression of CD31. (**b**) Flow cytometry quantificationally evaluated the expression of CD44^high^, CD90^high^, CD34^low^, and CD45^low^. Blue: fresh group; Green: LHe group. (**c**) RT-qPCR analysis evaluated the relative expression of four hBMSCs genes (PPARy, Collagen I, Sox9, and ALP) after vitrification in LHe. There was no significant difference between group Fresh and LHe. (**d**) Vitrified hBMSCs retained the adipogenic ability, osteogenic ability, and chondrogenic ability. (**e**) The relative stained area illustrated the differentiation ability of fresh and vitrified hBMSCs. There was no significant difference between group Fresh and LHe. Scale bars: 200 µm.

The stemness and multilineage differentiation ability of hBMSCs post vitrification in LHe were confirmed further (Figure 6). The expression of the typical hBMSCs positive marker CD44 and the negative marker CD31 (endothelial differentiation marker) were evaluated by immunofluorescence staining. As presented in Figure 6a, there was no significant difference of expression of CD44 and CD31 between fresh and vitrified hBMSCs. Furthermore, CD44, CD90, CD34, and CD45 markers were analyzed quantitatively by flow cytometry. The data (Figure 6b) showed that vitrification in LHe had no significant impact on surface marker expression for hBMSCs. Additionally, as detected by RT-qPCR, the relative expression of the typical hBMSCs genes, γPPARY (adipogenesis), Collagen I (osteogenesis), SOX9 (chondrogenesis), and ALP (osteogenesis), was not impacted post-vitrification (Figure 6c). The functional stemness of hBMSCs was further evaluated by testing multilineage differentiation ability. The lipid droplets in adipocyte cells reflected the successful adipogenic differentiation. Calcific deposition of osteoblast-like cells illustrated the ability of osteogenic differentiation, while sulfated proteoglycan deposit formation indicated maintenance of chondrogenic differential ability (Figure 6d,e). Therefore, vitrification in LHe has no impact on the functional characteristics of hBMSCs. 

### 3.5. Pluripotency of Vitrified hESCs

**Figure 7:**
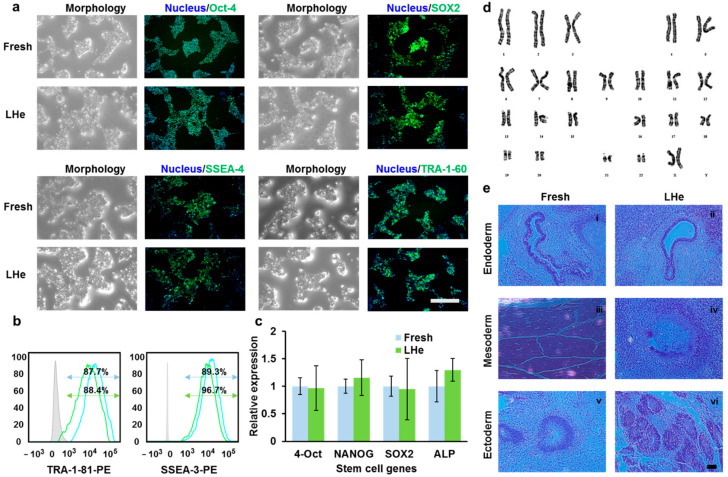
The hESCs retained pluripotency after vitrifying in LHe, indicated by the expression of typical genes and antigens, a normal karyotype, and teratomas formation. (**a**) Immunofluorescence staining indicated that vitrified hESCs showed stem cell markers: Oct-4, SOX2, SSEA-4, TRA-1-60. (**b**) Flow cytometry quantification of the expression of SSEA-3 and TRA-1-81 showed no significant difference between fresh (blue) and vitrified (green) hESCs. (**c**) The RT-qPCR analysis confirmed the relative expression of three hESC genes (4-Oct, NANOG, SOX2) was not influenced after vitrifying in LHe. There was no significant difference between group Fresh and LHe. (**d**) Vitrified hESCs retained a normal karyotype. (**e**) Pluripotency of hESCs vitrified in LHe. Fresh and vitrified hESCs developed into a teratoma when transplanted subcutaneously into SCID mice. Hematoxylin and eosin (H&E)-stained sections of (**i**,**ii**) respiratory epithelium (endoderm), (**iii**) muscle-like structure (mesoderm), (**iv**) cartilage-like structure (mesoderm), (**v**,**vi**) neuroepithelium (ectoderm). Scale bars: 200 µm.

**Figure 8:**
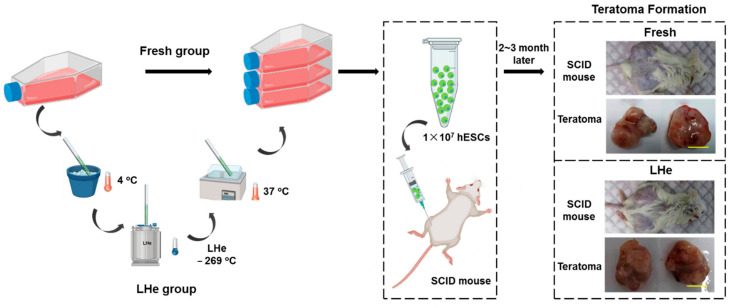
Schematic of teratoma formation for fresh and LHe-vitrified groups. Fresh or LHe-vitrified hESCs were cultured until the desired amount is achieved, then injected into SCID mice models. 2~3 months later, teratomas were fixed stained to confirm pluripotency of hESCs. Scale bar: 1 cm.

To confirm the functional characteristics of hESCs after vitrifying in LHe, the pluripotency of fresh cells and vitrified cells were investigated. As shown in Figure 7a, four typical hESCs markers, namely Oct-4, SOX2, SSEA-4, and TRA-1-60, were investigated by immunofluorescence staining. The data indicated that hESCs vitrified in LHe expressed normal stem cell markers as fresh hESCs. Additionally, flow cytometry analysis (Figure 7b) showed that the differentiation rate of hESCs vitrified in LHe was as low as that of fresh hESCs (SSEA-3: 87.7% vs. 88.4%; TRA-1-81: 89.3% vs. 96.7%). Three typical genes (4-Oct (also known as POU5F1), NANOG, and SOX2) expressing in the undifferentiated hESCs were explored by the RT-qPCR. The results indicated that vitrified hESCs showed no significant difference in the expression of the three genes (Figure 7c). In addition, hESCs vitrified in LHe showed a female molecular karyotype without aneuploidies (arr(1-22, x) × 2) (Figure 7d), which was consistent with the original data (https://discovery.lifemapsc.com/stem-cell-differentiation/in-vitro-cells/inner-cell-mass-homo-sapiens-line-h9-wa09-wicell-research-institute-inc, (accessed on 15 September 2021)). Non-vitrified and vitrified hESCs in LHe were injected into SCID mice to examine their pluripotency (Figure 8). The histologic analysis of teratomas proved that hESCs hold the ability to differentiate into three germ layers, indicated by respiratory epithelium (Figure 7(e(i),(ii)), endoderm), muscle-like structures (Figure 7(e(iii)), mesoderm), cartilage-like structures (Figure 7(e(iv)), mesoderm), and neuroepithelium (Figure 7(e(v),(vi)), ectoderm).

## 4. Conclusions

In this research, we first successfully vitrified hBMSCs and hESCs with high cell viability using LHe as the cryogen (for hBMSCs: 93.0 ± 0.7%; hESCs: 85.4 ± 4.9%; using high concentrations of CPAs). Importantly, even when decreasing the concentrations of CPAs, the comparative cell viability is still achieved by vitrifying in LHe (for hBMSCs: 85.2 ± 3.2%; hESCs: 79.0 ± 2.8%; using low concentrations of CPAs) compared with LN_2_ (for hBMSCs: 82.7 ± 4.1%; hESCs: 83.0 ± 2.0%; using high concentrations of CPAs). Moreover, both hBMSCs and hESCs post vitrification maintained high attachment efficiency, proliferation, stem gene expression, antigen expression, and intact multilineage differentiation properties. This high vitrification efficiency achieved by LHe was due to the high cooling rate, which was caused by the high transfer efficiency and the large temperature difference between LHe (−269 °C) and specimens. Therefore, LHe is expected to be applied in the field of vitrification and to promote the wide application of stem cell-based therapy.

## Figures and Tables

**Table 1:** Solutions for vitrification of hBMSCs and hESCs.

Cell Types	Equilibration Solution		Vitrification Solution	
hBMSCs	EFS15	15%EG-DPBS containing 20% FBS	EFS30	30%EG + 18% Ficoll 70 + 0.3 sucrose-DPBS containing 20% FBS
	EFS17.5	17.5%EG-DPBS containing 20% FBS	EFS35	35%EG + 18% Ficoll 70 + 0.3 sucrose-DPBS containing 20% FBS
	EFS20	20%EG-DPBS containing 20% FBS	EFS40	40%EG + 18% Ficoll 70 + 0.3 sucrose-DPBS containing 20% FBS
hESCs	EDS15	7.5%EG + 7.5%DMSO-culture medium	EDS30	15%EG + 15%DMSO + 0.5M sucrose-culture medium
	EDS17.5	8.75%EG + 8.75%DMSO-culture medium	EDS35	17.5%EG + 17.5%DMSO + 0.5M sucrose-culture medium
	EDS20	10%EG + 10%DMSO-culture medium	EDS40	20%EG + 20%DMSO + 0.5M sucrose-culture medium

Note: EG: ethylene glycol; DMSO: Dimethyl sulfoxide; DPBS: Dulbecco’s Phosphate Buffered Saline; FBS: fetal bovine serum.

**Table 2:** Typical antibodies to confirm hBMSCs and hESCs.

Cells	Antigens	Primary Antibody	Secondary Antibody
hBMSCs	Surface antigens	Rabbit anti-CD44 (Abcam) (ab189524)	AF 488 Goat anti-rabbit (Abcam) (ab150077)
		Mouse anti-CD31 (Abcam) (ab24590)	AF 488 Goat anti-mouse (Abcam) (ab150113)
hESCs	Intranuclear antigens	Rabbit anti-Oct-4 (Signalway Antibody) (#49129)	Mouse anti-rabbit IgG FITC (Bioss Antibodies) (bs-0295M-FITC)
		Mouse anti-SOX2 (Santa cruz Biotechnology) (sc-365823)	Anti-mouse IgGκ BP-FITC (Santa cruz Biotechnology) (sc-516140)
	Surface antigens	Mouse anti-SSEA-4 (Santa cruz Biotechnology) (sc-21704)	
		Mouse anti-TRA-1-60 (Santa cruz Biotechnology) (sc-21705)	

**Table 3:** The corresponding primers of stem and housekeeping genes used in RT-qPCR studies.

Cell Types	Gene	Primer	
hBMSCs	PPARγ	Sense primer	5′-TCT CTC CGT AAT GGA AGA CC-3′
		Anti-sense primer	5′-GCA TTA TGA GAC ATC CCC AC-3′
	Collagen type I	Sense primer	5′-GGG CAA GAC AGT GAT TGA ATA CA-3′
		Anti-sense primer	5′-GGA TGG AGG GAG TTT ACA GGA A-3′
	Sox9	Sense primer	5′-GCA GAG ACT GAA GAC CCT ACA CAG A-3′
		Anti-sense primer	5′-GAG GCA ACT TCA CGC TGC AA-3′
	ALP	Sense primer	5′-GCA AGA GCA CAA AGA GAA GAG-3′
		Anti-sense primer	5′-AAG GGG TCT ACA TGG CAA CT-3′
	GAPDH	Sense primer	5′-GCA AGA GCA CAA AGA GAA GAG-3′
		Anti-sense primer	5′-AAG GGG TCT ACA TGG CAA CT-3′
hESCs	Oct-4	Sense primer	5′-CTTGCTGCAGAAGTGGGTGGAGGAA-3′
		Anti-sense primer	5′-CTGVCAGTGTGGGTTTCGGGCA-3′
	NANOG	Sense primer	5′-CAACTGGCCGAAGAATAGCAATGGTAT-3′
		Anti-sense primer	5′-AAGGCAAGTCAGCAGCCATCTCAT-3′
	SOX2	Sense primer	5′-GCCTGGGCGCCAAGTAGA-3′
		Anti-sense primer	5′-GAACGAGCCGTTCATGTAGTCTG-3′
	GAPDH	Sense primer	5′-AGCCACATCGCTCAGACACC-3′
		Anti-sense primer	5′-AAGCTCTGTGGGACCTCTTG-3′

## Data Availability

The datasets generated and/or analyzed during the current study are not publicly available due to privacy policy but are available from the corresponding author on reasonable request.
